# New insight into the importance of formulation variables on parenteral growth hormone preparations: potential effect on the injection-site pain

**DOI:** 10.3389/fendo.2022.963336

**Published:** 2022-10-03

**Authors:** Bita Taghizadeh, Mahmoud Reza Jaafari, Nosratollah Zarghami

**Affiliations:** ^1^ Department of Medical Biotechnology, School of Advanced Medical Sciences, Tabriz University of Medical Sciences, Tabriz, Iran; ^2^ Biotechnology Research Center, Pharmaceutical Technology Institute, Mashhad University of Medical Sciences, Mashhad, Iran; ^3^ Department of Pharmaceutical Nanotechnology, School of Pharmacy, Mashhad University of Medical Sciences, Mashhad, Iran; ^4^ Department of Clinical Biochemistry and Laboratory Medicine, Faculty of Medicine, Tabriz University of Medical Sciences, Tabriz, Iran

**Keywords:** biopharmaceutics, patient adherence, patient compliance, subcutaneous, excipient, injection pain, growth hormone deficiency, formulation

## Abstract

Reducing injection-site pain (ISP) in patients with chronic conditions such as growth hormone deficiency is a valuable strategy to improve patient compliance and therapeutic efficiency. Thus understanding different aspects of pain induction following subcutaneous injection of biotherapeutics and identifying the responsible factors are vital. Here we have discussed the effects of formulation’s viscosity, concentration, osmolality, buffering agents, pH, and temperature as well as injection volume, dosing frequency, and different excipients on ISP following subcutaneous injection of commercially available recombinant human growth hormone products. Our literature review found limited available data on the effects of different components of parenteral rhGH products on ISP. This may be due to high cost associated with conducting various clinical trials to assess each excipient in the formulation or to determine the complex interactions of different components and its impact on ISP. Recently, conducting molecular dynamics simulation studies before formulation design has been recommended as an alternative and less-expensive approach. On the other hand, the observed inconsistencies in the available data is mainly due to different pain measurement approaches used in each study. Moreover, it is difficult to translate data obtained from animal studies to human subjects. Despite all these limitations, our investigation showed that components of parenteral rhGH products can significantly contribute to ISP. We suggest further investigation is required for development of long acting, buffer-free, preservative-free formulations. Besides, various excipients are currently being investigated for reducing ISP which can be used as alternatives for common buffers, surfactants or preservatives in designing future rhGH formulations.

## Graphical Abstract

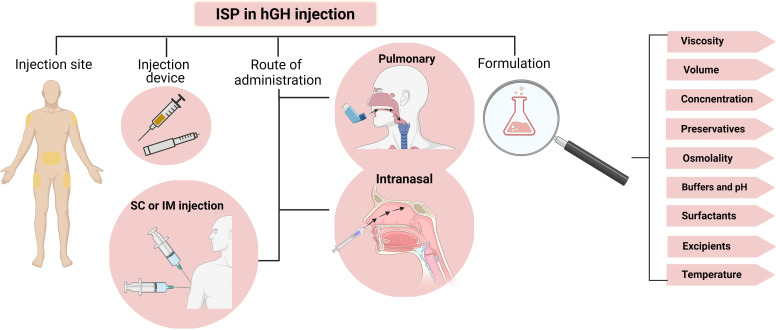

## Introduction

**Figure 1:**
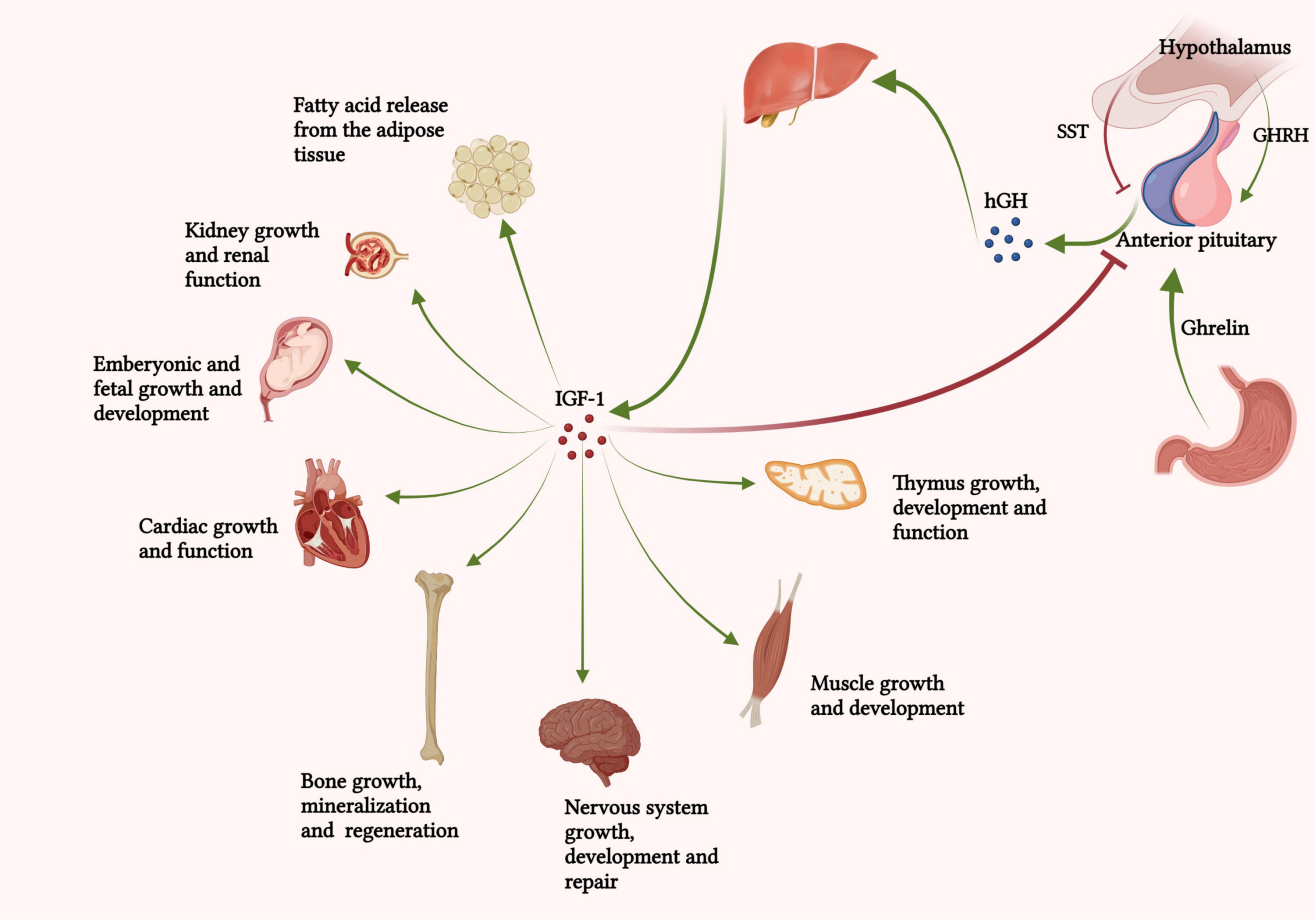
Growth hormone secretion and actions in the human body. “Created with BioRender.com.” Growth hormone secretion from the anterior pituitary gland is primarily regulated by hypothalamic SST and GHRH, ghrelin, and IGF-1. Secretion of hGH and its subsequent interaction with GHRs on the surface of hepatocytes promotes IGF-1 secretion. “Created with BioRender.com”.

Human growth hormone (hGH or somatotropin) is a 22 kDa, single-chain peptide with 191 amino acids, two disulfide bonds (Cys53-Cys165, Cys182-Cys189), and four alpha helixes (1). Human growth hormone is produced by the somatotroph cells of the anterior pituitary gland and is released *via* 4-8 hormonal bursts of 0.5-0.8 mg each day (2). The pulsatile secretion of GH is responsible for its metabolic and anabolic effects. **Figure 1** summarizes the most important physiological functions of hGH in the human body. The extensive contribution of GH in somatic growth and maintaining hemostasis *via* a broad range of biochemical processes during childhood, adulthood, and adolescence, implies its importance in the management of different disorders.

Between 1963 and 1985 nearly 35000 children with growth hormone deficiency (GHD) received hGH extracted from the cadaver’s pituitary gland (3). Since the pituitary gland of cadavers was the only resource for obtaining hGH at that time, only children with severe GHD could receive hGH treatment. The development of recombinant hGH (rhGH) provided an unlimited source of hGH to meet the high demand and compensate for limited sources of the hormone. The first rhGH became available by Genentech in 1979 (4), and large quantities of purified rhGH became accessible for the first time. Genentech’s rhGH (Protropin^®^) was approved by the Food and Drug Administration (FDA) in 1985 and became the second recombinant drug (insulin being the first) to be developed and manufactured by a biotechnology company. Since then, rhGH has been the focus of interest for biotech and pharmaceutical companies, and several rhGH-based biopharmaceuticals have been introduced for the management of different disorders (**Table 1**).

**Table 1:** Current hGH indications.

Pathological condition		References	Pathological condition		References
**Growth hormone deficiency**		(5–7)	**Surgical trauma**		(8, 9)
**Prader-Willi syndrome**		(8)	**Sepsis**		(10, 11)
**Turner syndrome**		(12, 13)	***In vitro* fertilization (IVF)**		(14, 15)
**Small for gestational age**		(16, 17)	**Fibromyalgia**		(18, 19)
**Noonan syndrome**		(20, 21)	**Skeletal dysplasia**		(22, 23)
**Idiopathic short stature**		(5, 24)	**Muscle-wasting and fat accumulation secondary to HIV**		(10, 25)
**Chronic renal failure**		(26, 27)	**Inflammatory bowel disease**		(28, 29)
**Arthritis**		(30–32)	**Cystic fibrosis**		(33–35)
**Cachexia**		(36, 37)	**Short bowel syndrome**		(38)

Growth hormone deficiency involves children and adults and can be congenital (due to genetic defects or perinatal injuries) or acquired (due to pituitary and non-pituitary tumors, radiotherapy, or severe head injury). Recombinant growth hormone replacement therapy is a routine treatment for children and adults with GHD. Due to the short plasma half-life of hGH (0.36 and 3-4 hours after subcutaneous (SC) and intravenous (IV) injections), frequent injections are necessary (39). Once GHD is diagnosed, rhGH replacement therapy should begin as soon as possible. The most common complications associated with untreated GHD include neurological, cardiovascular, skeletal, and metabolic disorders with a higher chance of premature death (40, 41).

Although the duration of treatment is still under debate, the treatment usually lasts for several years. Due to the chronic nature of the condition, there is a strong demand for novel formulations with higher stability, more straightforward application, and enhanced patient convenience to improve therapeutic effectiveness *via* improving patient adherence. The ideal parenteral rhGH preparation should remain stable and effective during product manufacturing and storage, induce no adverse effects or immune responses, and induce minimum pain and discomfort upon injection while being easy to use. To meet this need, different injectable formulations, delivery systems, injection devices, and routes of administration have been introduced in the last few years.

The current review discusses the effect of parenteral rhGH formulations’ characteristics on their therapeutic efficacy regarding patient adherence (specifically the injection site pain). Here we have discussed the effects of formulation’s viscosity, concentration, osmolality, buffering agents, pH, and temperature as well as injection volume, dosing frequency, and different excipients on ISP following subcutaneous injection of commercially available rhGH products.

## Injection-site pain

According to the International Association for the Study of Pain (IASP) definition, pain is an unpleasant experience with physiological or psychological routes, accompanying or resembling tissue damage (42). Due to the complex nature of the phenomenon, measuring pain intensity has always been a challenge for scientists conducting clinical trials. In this regard, three main approaches have been developed including visual analogue scale (VAS), numeric rating scale (NRS), and verbal rating scale (VRS) for measurement of pain intensity. In VAS subjects are asked to mark their pain severity on a 10 cm long line. The left- and right-hand sides of this line represent “no pain” and “worst pain”, respectively. The markings are then converted to a numerical scale of one to 10. NRS is a scoring system that asks subjects to give a score to their pain on a scale of one to 10 (one being “no pain” and 10 being “worst pain”). In contrast to the other two methods, VRS uses a verbal scale for measuring pain severity and asks subjects to describe their pain as “none”, “light”, “moderate” or “severe”. Among these, NRS has shown superior compliance and sensitivity compared to the other two approaches (43).

ISP can be affected by the characteristics of the formulation or the injection device. Needle size, length, and the angle of injection are the mechanical features of injection devices determining the degree of ISP in SC injection. Different self-injecting needle pens and needle-free devices have been developed for more straightforward injection, accurate dosing, electronic monitoring, and injection pain reduction (44–48).

Parenteral rhGH formulations are carefully designed preparations containing different excipients such as buffering agents (phosphate, citrate, histidine, glycine, carbonate, and acetate), tonicity adjusting excipients (mannitol and sodium chloride), bulking agents (sugars, polyols, amino acids, and polymers), surfactants (polysorbate 20 and poloxamer 188) and preservatives (antimicrobial agents and chelators). These excipients are necessary to provide stability and maintain hGH functionality during manufacturing and storage and prevent microbial contamination. ISP can be affected by various formulation features, including concentration, osmolality, viscosity, pH, injection volume, preservatives, and buffers (49). In the following sections, we will discuss the effect of each characteristic on the ISP following SC injection of hGH.

## Patient adherence

Patient adherence is a critical challenge in managing chronic conditions such as GHD that can limit the effectiveness of rhGH replacement therapy, especially during the first two years of starting the treatment (50). Based on patients’ age, adherence to treatment regimen decreases in the following order: adulthood>childhood>adolescence (51, 52). Patient adherence becomes more challenging when the drug should be administered *via* injection. As for hGH therapy, it has been shown that patient non-adherence varies from 5 to 82% (53). A study by Smith and colleagues reported that 50% of growth hormone deficient children failed to comply with their treatment regimen (54).

The SC route is preferred to intramuscular (IM) injection for biopharmaceuticals by both patients and physicians due to lower pain, the possibility of at home-administration, and lower cost burden for patients who need to meet their physicians regularly and receive their hGH injections in medical facilities, all of which favor patient convenience (55). No difference has been observed in the growth-promoting effect of hGH between SC and IM injection (56). However, the absorbance profile and bioavailability of SC- and IM-injected hGH has shown inconsistencies. For example, hGH (1.3 mg/m^2^/day) reaches its peak plasma concentration (T_max_) at 2 and 4 hours and returns to its baseline after 9 and 18 hours following IM and SC injections, respectively (57, 58). Accordingly, the available time for absorption of hGH following IM injection is not long enough to provide physiologically-relevant plasma levels by daily administration. The longer retention time of the biopharmaceuticals at the SC injection site provides extended drug release compared to IM injection. Rapid drug absorption upon IM injection is due to the high vasculature of the muscle tissue (59).

Another study by Russo and colleagues showed that the peak plasma concentration (C_max_), T_max_, and antibody responses were similar for SC and IM injections of hGH (0.03 mg/kg/day) in children with GHD. In contrast, SC injection yielded a larger integrated hGH concentration. Patient acceptance and compliance were also significantly higher for SC injection since it was less painful. Their results concluded that the SC route is safe, efficient, and well accepted by the patients for hGH administration (56). The higher pain sensation associated with the IM route is related to the higher number of nerve fibers in the muscle than in SC tissue (60). In general, water-soluble, low molecular weight, and low viscosity molecules in near-neutral pH are ideal for SC injection (61). In addition to injection pain, ease of use is another determining factor affecting adherence to growth hormone replacement therapy (62).

Increased SC injection frequency yields more physiologically-relevant results compared to 2-4 times/week IM injections (56, 63). However, the pain associated with frequent SC injections renders patients unwilling to adhere to their treatment, especially in children who comprise most growth hormone deficient patients (64). According to a study by Liedert et al., pain was the most frequent adverse effect following SC injection of liquid and lyophilized hGH formulations, reported by 87% of the tested subjects (65).

Patient convenience can be improved by reducing the injection pain or frequency or changing the administration route. The injection frequency can be reduced by using long-acting formulations with sustained rhGH release (66–68). In addition, different non-invasive routes for growth hormone administration have been suggested, including intranasal, transdermal, and pulmonary (69–76).

The frequency of injections and necessary preparation steps before injection can significantly influence patients’ compliance (77). For example, it has been shown that the required reconstitution of rhGH before the injection is a significant factor limiting patient compliance (54). Liquid and freeze-dried rhGH formulations are both used for hGH replacement therapy. According to a study by Muller et al., the need for the reconstitution of the freeze-dried form (Norditropin^®^) before injection implies a significant negative impact on patient compliance. In their research, the solution form (Norditropin^®^ SimpleXx^®^) was preferred to the powder form due to its easier application. Their results suggest that the level of necessary reconstitution before use should be considered in the development of the formulations for improving compliance (78).

In another study, rhGH solution (Norditropin^®^ SimpleXx^®^) was preferred by 85% of the patients to the freeze-dried powder form due to improved handling. Assessment of pain perception at the injection site showed that 35% of patients found the liquid form less painful, and 59% reported similar pain intensity as the powder form. For 6% of patients, the liquid form was more painful (66). The acceptability and patient compliance were studied in another clinical trial on 53 patients with GHD (adults and children) who used Norditropin^®^ SimpleXx^®^ for 6 weeks. According to 90% of the patients, easier handling, no reconstitution before injection, and decreased pain perception following Norditropin^®^ SimpleXx^®^ injection were superior to the conventional powder form (79). Stanhope and colleagues studied the acceptability of liquid injectable rhGH form in 103 children with GHD for 12 weeks. About 92% of these patients preferred Norditropin^®^ SimpleXx^®^ and reported easier application and decreased pain sensation following SC injection (80).

The acceptability of four different commercially available rhGH formulations (Norditropin SimplexX, Humatrope, Genotropin, and Nutropin AQ) was evaluated in 109 pediatric patients with GHD in an open-label, randomized, multicenter, cross-over study for one month (81). Accordingly, Norditropin was preferred to Humatrope, Genotropin, and Nutropin by 77%, 71%, and 94% of the patients, respectively. The patient’s parents preferred the Norditropin cartridge over the other three rhGH products due to easier preparation, dosing, and application.

### Viscosity, injection volume, and concentration

Protein solution viscosity is an important consideration in developing liquid biopharmaceutical formulations. Injectable formulations with high viscosity often face issues of low protein stability and poor flow features. Various features of the protein including shape, size, charge distribution, concentration, and association kinetics are involved in determining the viscosity of the solution. On the other hand, these molecular features are affected by solution conditions such as temperature, ionic strength, additives, and pH (82). Furthermore, attractive (charge-dipole, charge-charge, Van der Waals, and hydrophobic) and repulsive (charge-charge, and exclude volume effect) forces are also involved in determining the viscosity of a protein solution (83). Taken together, predicting the viscosity of a protein solution is not an easy task due to the involvement of complex inter- and intra-molecular interactions, protein properties, as well as solution’s condition. Instead, controlling the biopharmaceutical solution’s viscosity is rather easier. In general, the higher the protein concentration, the higher is the formulation’s viscosity (84). However, the increase in viscosity is not always linear with regard to protein concentration (82).

The role of viscosity in ISP and its possible underlying mechanism have not been thoroughly investigated. However, Schwarzenbach et al. showed that the solution’s viscosity had a significant effect on ISP. Subcutaneous injection of solutions with low (1 cP) and medium viscosity (8-10 cP) were found to be more painful compared to highly viscose (15-20 cP) solutions (85). Their results showed that an SC injection< 3 mL with 15-20 cP viscosity is well tolerated (86).

Viscosity-reducing gents such as NaCl and amino acids are commonly added to formulations as tonicity adjusting agents to reduce ISP since injection of hyper- or hypotonic parenteral formulations can induce cell shrinkage or swelling leading to increased pain (87). However, NaCl concentration has been linked to ISP in formulations with acidic pH (5.7 compared to 6.5). Increasing NaCl concentration in 5 mM histidine buffer at pH 5.7 from 25 to 75 mM was accompanied by increased ISP. However ISP in a higher NaCl concentration (75 mM) in acidic, but near physiologic pH of 6.7 was less significant than a lower NaCl concentration of 25 mM in more acidic pH (5.7) (88).

Higher injection volumes are associated with higher ISP and the maximum volume of 2 mL per dose is recommended (1.5 mL is common). In this regard, it has been shown that SC injection of smaller volumes of rhGH preparations is less painful and improves patient convenience (89–91). In a study by Chantelau et al., ISP was unrelated to the injection volume at volumes ≤ 0.5 mL (92). Another study evaluated the severity of pain inflicted by SC injection of different volumes of 0.9% NaCl solution (0.2, 0.5, 1, and 1.5 mL). The results showed that an injection volume in the range of 0.5-1 mL is directly related to the severity of ISP (93). According to these results, injection volumes ≤ 1.0 mL are preferred, and 0.5-0.8 mL is ideal (94–96).

One approach to reducing the injection volume is to increase hGH concentration. However, as mentioned previously, highly concentrated hGH formulations have higher viscosity and thus a higher risk of aggregate or insoluble particulate formation, compromising product stability and safety. This finding suggests the confounding effect of protein concentration and formulation’s viscosity on ISP. Hansen and colleagues studied the effect of hGH concentration and injection volume on ISP and concluded that hGH concentration was directly associated with pain perception following SC injection. They observed that injection of 3 mg/300 µL of hGH solution was more painful compared to 1 mg/300 µL solution. Consistent with previous studies, they observed that increasing the injection volume from 300 to 600 µL while maintaining hGH concentration (2 mg) caused more pain (97).

The injection volume is determined based on product concentration and dosing regimen. Daily hGH dosing is highly patient-specific (depending on the patient’s age, disease, and comorbidities) and can be determined according to weight-based or non-weight-based strategies. For example, The recommended daily dose for treatment of GHD, according to patient’s age and non-weigh-based approach, is as follows; 0.4-0.5 mg/day for patients ≤ 30 years old, 0.25 mg/day for patients between 30 and 60 years old and 0.15 mg/day for patients≥60 (98). Hence the average dose for adults with GHD is estimated to be 0.3 mg/day. With this amount of hGH dosage, the injection volume is not expected to affect ISP. It should be noted that the initial dose for GHD patients is gradually increased by 0.1-0.2 mg/day increments after each month. However, the daily hGH dose does not generally exceed the maximum of 2 mg/day for these patients. Higher doses of 6 and 8 mg/day might be administered for adults with cachexia and short bowel syndrome, respectively. In this case, higher injection volumes might be needed.

### Dosing frequency and antimicrobial preservatives

Low stability and relatively short plasma half-life of rhGH demand frequent injections (99). In addition, it has been shown that daily hGH injection has a superior growth-promoting effect compared to administering the same dose over 2-4 injections per week (54, 63). However, it can be expected that patient non-adherence will increase with dosage frequency due to repeated ISP. McNamara et al. confirmed this by showing that GHD patients prefer treatment plans involving less frequent hGH injections (100).

Parenteral rhGH products are available as single- or multi-dose preparations. The single-dose formulations are used for a single injection in a single patient. Due to the disposable nature of these preparations, they usually do not contain antimicrobial preservatives. On the other hand, multi-dose vials or cartridges can be used more than once and require additional preservatives to prevent microbial contamination after the first use. Antimicrobial preservatives have been associated with ISP in parenteral biopharmaceuticals, including rhGH products. Phenol, *m*-Cresol, and benzyl alcohol are the most commonly used preservatives in multi-dose rhGH products.

According to research conducted on 197 patients with GHD who received SC rhGH injections for one year, *m*-Cresol was associated with higher local pain compared to 0.9% benzyl alcohol (101). In another study, Bridges and colleagues performed a double-blinded, randomized, cross-over trial on 31 children to compare pain sensation following SC injection of rhGH formulations reconstituted with benzyl alcohol (0.9 and 1.5%), *m*-Cresol (0.25%), or benzyl alcohol (0.9%)+glycerol (0.1%). Based on their results, the *m*-Cresol injection was more painful compared to benzyl alcohol, while no significant difference was observed between injection of 0.9% and 1.5% benzyl alcohol preparations (102). Subcutaneous injection of phenol (4.5 mg/mL) has been associated with less pain compared to benzyl alcohol (15 mg/mL) (103).

A case report by Bach and colleagues showed immunological reaction and myalgia development following SC injection of an *m-*Cresol-preserved rhGH product (Humatrope^®^) (104). Similar reactions were previously reported for the *m-*Cresol component of commercial insulin as well (105). Another study by Svendsen and Carstensen showed local toxic effects of high concentrations of benzyl alcohol, *m*-Cresol, and phenol upon 1 mL IM injection in rabbits. According to their results local toxicity increases in the following order; benzyl alcohol<phenol<*m*-cresol. Benzyl alcohol showed the least toxicity and only in concentrations above 15 mg/mL, while this was observed at lower concentrations of 7.5 and 3 mg/mL for phenol and *m*-cresol, respectively (106). Based on these data, it can be concluded that m-cresol injection induces more pain and local toxicity compared to phenol and benzyl alcohol.

Another strategy for reducing the ISP is to add a local anesthetic agent to the parenteral formulation (60). The local anesthetic effect of benzyl alcohol upon SC injection has been shown previously (107, 108). St Peter and colleagues compared ISP following SC injection of single- and multi-dose Epogen^®^. According to their results, SC injection of benzyl alcohol-containing multi-dose formulation was less painful compared to the benzyl alcohol-free single-dose form (109). In a series of three randomized, double-blinded, cross-over trials on the ISP for different QS-21 adjuvant formulations, it was observed that the addition of benzyl alcohol (0.72%) to QS-21 formulation substantially reduced pain upon IM injection (110). It can be expected that the anesthetic effect of benzyl alcohol may be, in part, responsible for the observed lower ISP.

### Osmolality

The ideal osmolality for isotonic subcutaneously injectable solutions is 300 mOsm/kg (285-295 mOsm/kg) (111). Injection of hypertonic preparations has been associated with increased local pain and discomfort (112, 113). However, manufacturing companies might prefer hypertonic preparations to reduce the injection volume or to maintain protein stability/solubility. The maximum osmolality of 600 mOsm/kg is relatively tolerable for SC injections ≤ 0.5 mL (114).

### Buffers and pH

The ideal rhGH formulation should be prepared with near physiological pH (111). However, balancing protein’s stability/solubility while maintaining solutions’ near-neutral pH can be challenging. Buffers are commonly used for maintaining the pH in biopharmaceutical formulations. The buffering capacity of a buffering agent depends on different factors, including pKa, the solution’s pH, and buffer concentration (115). Buffer type, strength, and concentration affect the local pain following SC injection. **Table 2** presents commonly used buffering agents in parenteral rhGH products. As shown previously, subcutaneous injection of citrate buffer is more painful than normal saline and phosphate buffer (116–118).

**Table 2:** Commonly used buffers in rhGH products, their concentration range and buffering capacity.

Buffer	pH range	pKa
Phosphate	3.0-8.0	2.1, 7.2 and 12.3
Citrate	2.1-6.2	3.1, 4.8 and 6.4
Glycine	8.8-10.8	2.3-9.7
Acetate	3.8-5.8	4.8
Tromethamine	7.0-9.0	8.1
Carbonate	7-8	6.3-10.3
Histidine	5.0-6.5	1.8, 6.1 and 9.2
Succinic acid	4.3-6.6	4.2 and 5.6

It has been established previously that infusion of acidic formulations is painful. The difference between the pH of the formulation and the injection-site tissue is responsible for pain sensation. The increased number of H ions upon SC injection of a formulation with an acidic pH compared to the physiological pH of the injection-site tissue activates nociceptors, which are responsible for pain sensation upon SC injection of preparations with non-physiological pH (119). Yang and Lai have recently provided mechanistic insight regarding the contribution of acids and citrate in ISP (120). They showed how acids stimulate and citrate potentiates acid-sensing ion channel 1 (ASIC1). These findings can explain painful injections of slightly acidic formulations containing low citrate concentrations. They suggested that the addition of ASIC1 inhibitors to citrate-containing preparations can decrease ISP without the need to eliminate citrate. Since ASIC1 potentiation by citrate involves extracellular calcium ion chelation, they proposed supplementing the formulation with Ca^2+^ can prevent pain induction in citrate-buffered preparations (120). In addition to direct activation of ASIC by H^+^ ions, research has shown that exposure of afferent nerves to acidic solutions with pH<6 can also induce pain *via* indirect activation of ASIC (121).

The manufacturers might prefer to produce parenteral protein formulations in non-physiological pH due to stability issues. In this case, using buffers with lower strength is recommended to decrease the ISP. In a study by Fransson et al., different phosphate buffered biopharmaceuticals with different pH values were compared regarding ISP following SC injection (122). The tested preparations included isotonic hIGF-1 with 5-50 mM phosphate buffer and pH 6.0-7.0. Among these, 10 mM phosphate buffer and pH 7.0 induced the lowest pain. They showed that increasing buffer concentration at non-physiological pH (50 mM phosphate and pH 6.0) substantially increased ISP (122). To minimize the ISP, it has been suggested that the maximum concentration of citrate and phosphate buffers in parenteral preparations should be limited to 7.3 and 10 mM, respectively (123).

As mentioned previously, the addition of strong buffers (such as citrate) to the formulation will increase pain due to radical pH changes within the SC tissue following injection (124). Accordingly, it has been observed that low-strength buffers (such as histidine) are less painful compared to phosphate and citrate in the SC injection of rhGH formulations (103). Laursen and colleagues studied the dispensing solutions from two commercially available parenteral rhGH products regarding ISP following SC injection (103). Norditropin^®^ SimpleXx^®^ and Nutropin AQ^®^ use histidine and citrate as their buffering agents, respectively. In this study, 54 healthy volunteers were injected with 0.3 mL of three test solutions as follows; A) 1.36 mg L-histidine, 6 mg Phenol with pH=6.15, B), 10 mM Na-Citrate, 5 mg Phenol with pH=6 and C) 0.9% normal saline, 9.0 mg benzyl alcohol with pH=8.3. They observed that the citrate-buffered formulation induced higher ISP, while the histidine-buffered formulation did not imply more pain compared to normal saline. They suggested that histidine is superior to citrate in reducing the ISP of rhGH injection (103). Meanwhile, Shi and colleagues reported that ISP following SC injection of citrate- and histidine-buffered formulations were not significantly different (88). It should be noted that, unlike Laursen et al., the formulations tested by Shi and colleagues were prepared without the bioactive agent (i.e., the protein).

In addition, citrate concentration has been directly associated with the severity of injection pain. Shi and colleagues showed that SC injection of 20 mM citrate solution was more painful compared to 5 and 10 mM solutions (88). They also reported that the injection of histidine buffer with a slightly acidic pH (6.5) was less painful compared to a more acidic histidine solution (pH 5.7) (88), which is in accordance with other studies suggesting pH deviation of parenterals from the neutral condition can induce pain at the site of injection.

As presented in **Table 3** the pH of commercial parenteral rhGH products ranges from 5.8 to 9.0. The liquid rhGH dosage forms are formulated at slightly acidic conditions compared to the freeze-dried forms. Based on a study by Ward et al., SC injection of an alkaline albumin formulation (pH 10) was associated with increased pain and discomfort at the injection site. However, the reported discomfort was described as slight to moderate despite the significant pH difference between the albumin preparation and SC tissue (125). They suggested that the low concentration of glycine buffer (20 mM) was responsible for minimizing the ISP despite the significant pH difference between the SC tissue and the parenteral albumin (125). As mentioned previously, using weak buffers is recommended in formulations with non-physiological pH. They suggested that the low glycine concentration minimized the ISP by allowing the rapid pH change of albumin preparation towards the physiological pH of the SC tissue upon injection (125).

**Table 3:** FDA-approved rhGH products for subcutaneous administration.

Company	Brand	Dosage Form	Buffer	pH	Preservative	Concentration	Other Excipients
**Pfizer**	Genotropin^®^	Powder	Phosphate	6.7	*m*-Cresol	5 and 12 mg/ml	Glycine Mannitol Sodium dihydrogen phosphate anhydrous Disodium phosphate anhydrous
	Genotropin^®^ MiniQuick	Powder	Phosphate	6.7	Preservative-free	0.2, 0.4, 0.6, 0.8, 1, 1.2, 1.4, 1.6, 1.8 and 2 mg/ml	Glycine Mannitol Sodium dihydrogen phosphate anhydrous Disodium phosphate anhydrous
**Novo Nordisk**	Sograya^®^ (Sustained-release)	Solution	Histidine	6.8	Phenol	6.7 mg/ml	Mannitol Phenol Poloxamer 188
	Norditropin^®^	Solution	Histidine		Phenol	5 mg/1.5 ml, 10 mg/1.5 ml, 15 mg/1.5 ml and 30 mg/1.5 ml	Poloxamer 188 Mannitol
**Genentech**	Nutropin^®^	Powder	Phosphate	7.4	Benzyl alcohol	5 and 10 mg/ml	Glycine Mannitol Sodium phosphate monobasic Sodium phosphate dibasic
	Nutropin AQ^®^	Solution	Citrate	6	Phenol	5, 10 or 20 mg/ml	Polysorbate 20 NaCl
	Nutropin Depot^®^ (long-acting)	Powder	Acetate and carbonate	5.8-7.2	Preservative-free	13.5, 18 and 22.5 mg/3ml	Zinc acetate Zinc carbonate PLG Polysorbate 20 Carboxymethylcellulose sodium salt NaCl
	Somatrem^®^ (Protropin^®^)	Powder	Phosphate	7.4	Benzyl alcohol	5 mg/ml	Mannitol Sodium phosphate
**Sandoz**	Omnitrope^®^	Solution	Phosphate	7	Benzyl alcohol	5 mg/1.5 ml cartridge	Disodium hydrogen phosphate heptahydrate Sodium dihydrogen phosphate dihydrate Poloxamer 188 Mannitol
					Phenol	10 mg/1.5 ml cartridge	Disodium hydrogen phosphate heptahydrate Sodium dihydrogen phosphate dihydrate Poloxamer 188 Glycine
		Powder	Phosphate	7	Benzyl alcohol	5.8 mg/vial	Disodium hydrogen phosphate heptahydrate Sodium dihydrogen phosphate dihydrate Glycine
**EMD Serono**	Saizen^®^	Powder	Citrate	6.5-8.5	Benzyl alcohol	5 mg/vial	Sucrose O-phosphoric acid
						8.8 mg/vial	Sucrose O-phosphoric acid Glycine
	Serostim^®^	Powder		6.5-8.5	Preservative-free	5 mg single-use vials	Sucrose Phosphoric acid
				7.4-8.5	Preservative-free	6 mg single-use vials	
				7.4-8.5	Benzyl alcohol	4 mg multiple-use vial	
	Easyclick^®^	Powder	Citrate	6.5-8.5	*m*-Cresol	5.83 mg/ml	Sucrose Phosphoric acid
	Zorbtive^®^	Powder	Phosphate	7.4-8.5	Benzyl alcohol	8.8 mg/vial	Sucrose Phosphoric acid Sodium phosphate dibasic Glycine
**Biotechnology General**	Biotropin^®^	Powder			Benzyl alcohol	3.33 and 4 mg/vial	NaCl
			Citrate	5.5-6.5	Phenol	5.83 and 8 mg/ml cartridges	Sucrose Poloxamer 188 Citric acid
**Ferring**	Zomacton^®^	Powder		7-9	Benzyl alcohol	5 mg vial	Mannitol NaCl
			Phosphate		*m*-Cresol	10 mg vial	Mannitol Disodium phosphate dodecahydrate Sodium dihydrogen phosphate dehydrate
**Biopartners GmbH**	Valtropin^®^	Powder	Phosphate	7.5	*m*-Cresol	3.33 mg/ml	Glycine Mannitol Monobasic sodium phosphate Dibasic sodium phosphate
**Cangene**	Accretropin^®^	Solution	Phosphate	6	Phenol	5 mg/ml	Poloxamer 188 Sodium phosphate NaCl
**Eli Lilly**	Humatrope^®^	Powder	Phosphate	7.5	*m*-Cresol	5 mg/vial 6, 12 and 24 mg cartridges	Mannitol Glycine Dibasic sodium phosphate Glycerin
**TEVA**	TEV-Tropin^®^	Powder	Phosphate	7-9	Benzyl alcohol	5 mg/vial	Mannitol NaCl
					*m*-Cresol	10 mg/vial	Mannitol Sodium dihydrogen phosphate
**ASCENDIS PHARMA ENCOCRINOLOGY DIV A/S**	SKYTROFA^®^ (long-acting)	Powder	Tromethamine	5	Preservative-free	3, 3.6, 4.3, 5.2, 6.3, 7.6, 9.1, 11 and 13.3 mg/vial	Succinic acid Trehalose dihydrate

Perhaps the most comprehensive study regarding the effects of different buffering agents and excipients on ISP has been recently conducted by Shi and colleagues (88). They studied various buffered formulations with different concentrations and pH in combination with commonly used tonicity adjusting excipients. Their results confirmed the substantial effect of buffer on ISP. In line with previous studies, they also found citrate and histidine to increase ISP. Higher buffering capacity and concentrations were also associated with increased ISP. They suggested that the increased injection pain in acidic formulations containing strong buffers is the result of high H^+^ concentration at the injection site. These sustained protons can induce pain *via* activating ASICs and transient receptor potential ion channels, which is in line with the results reported by Yang et al. (88, 120, 126).

Since buffer strength and concentration are actively involved in pain sensation following SC injection of parenteral drugs, the development of buffer-free formulations can minimize ISP. In this regard, citrate-free Adalimumab formulation has been reported to be less painful compared to the conventional citrate-buffered preparation (120). Shi and colleagues also supported this by showing that citrate- and phosphate-free formulations were substantially less painful (88).

A study by Gharia and Sudhakar showed that SC injection of a succinate-buffered Adalimumab biosimilar was considerably less painful compared to the citrate-buffered form (127).

### Surfactants

Non-ionic surfactants are widely used in parenteral biopharmaceutical products to avoid protein aggregation. Tween 20 (polysorbate 20 or PS20) and poloxamer 188 (P188 or pluronic F68) are commonly used non-ionic surfactants in parenteral hGH products. Despite their protective role, both PS20 and P188 are susceptible to auto-oxidation producing reactive species that can induce protein degradation or injection-site reactions (ISR), including injection-site pain. Singh and colleagues suggested that PSs’ degradation in biopharmaceutical preparations can be responsible for ISRs following the administration of biologics. They suggested that using high-quality raw materials and optimizing shipping and storage conditions can minimize PSs-induced ISR (128). Jewell et al. studied the tolerability of poloxamer 188 injection in healthy volunteers and showed that pain and ISR were the most common side reactions upon P188 injection (129). In another study, Jung and colleagues compared ISP following IV injection of two different propofol formulations; LCT propofol (containing 1% Diprivan^®^) and Aquafol™ (reformulated micro-emulsion formulation containing 1% propofol, 10% P188, and 0.7% polyethylene glycol 660 hydroxystrease). They also observed that the injection of P188-containing micro-emulsified formulation was more painful (130). It has been suggested that sugar-based surfactants such as alkylglucosides can be used as alternatives to polysorbates (131).

### Other excipients

Non-isotonic formulations can increase ISP *via* activating stretch receptors (132). According to Shi and colleagues, the contribution of NaCl in the ISP is more pronounced compared to sugars and polyols (including sucrose, trehalose, and mannitol). NaCl-containing formulations were associated with increased ISP and the severity of ISP increased with NaCl concentration. They concluded that the sodium ions from NaCl were responsible for increased ISP and not the chloride ions, and suggested using Arginine-HCl instead of NaCl could reduce ISP (88).

Sorbitol and mannitol are two isomeric sugar alcohols used in rhGH products, providing stability and tonicity adjustment. As mentioned previously, incorporating excipients with local anesthetic effects has been associated with reduced ISP. The significant pain relief observed following the injection of highly concentrated sorbitol (4%) solutions is attributed to its antioxidant activity (133–135). In addition to the local anesthetic effect of mannitol in combination with lidocaine which has been shown previously (136, 137), antinociceptive properties of SC mannitol injection in synergism with diphenhydramine have also been reported recently (138).

### Temperature

Warming parenteral solutions before infusion is a well-known strategy for reducing injection pain as the infusion of preparations with room temperature (20-25°C) have been less painful (139). The rationale behind this phenomenon relies on the activating effect of a lower formulation temperature on the nociceptors following SC injection (140–142). Most biopharmaceuticals should be refrigerated at 2-8°C, while the SC tissue temperature is approximately 34°C. Allowing the parenteral product to reach room temperature before injection can reduce the ISP (143, 144).

## Discussion

Despite recent advances in the field of formulation development, limited or conflicting data are available in the literature on the effect of formulation components on ISP. In addition, the mechanisms by which each of the formulation components may contribute to injection pain remains elusive. Another issue arises from the fact that a large number of reports regarding injection pain are conducted on animals which makes it difficult to translate these findings to human subjects. Another problem with the existing studies that may in part explain their inconsistent results is using different approaches for quantifying and assessment of pain in tested subjects. The lack of comprehensive studies regarding the impact of different formulation variables on ISP may be due to the high cost associated with conducting various clinical trials for pharmaceutical companies and the difficulty in studying the complicated interdependent interactions of these formulation parameters on ISP and identifying the exact mechanism by which they affect injection pain.

This review aimed to explore the effect of different formulation variables on ISP in GHD patients. Our investigation suggests that complex interactions between the formulation variables should be considered in addition to the individual contribution of each variable in ISP. In some cases, such as protein concentration, formulation’s viscosity, and injection volume confounding effects were observed which require further investigation. In addition, we found no report in the literature on the effect of growth hormone concentration and formulation’s osmolality on ISP in parenteral rhGH products.

Formulation components in injectable preparations should be selected cautiously. For example, incompatibility between surfactants and other excipients in the formulation might promote surfactants’ degradation leading to increased ISP. In addition, using high-quality excipients with no residual contamination can inhibit protein and excipients degradation as well as subsequent ISR including injection pain. Using molecular dynamics simulation before formulation design has been suggested in a recent study for easier assessment of the interactions between different formulation components (145). More research is needed towards the development of buffer-free and preservative-free formulations. It has been recently suggested that the removal of phenolic preservatives from the commercially available insulin products before SC injection by using Z-Y filtration reduces the inflammatory reactions induced by repeated SC injections (146, 147). Finally, alternative buffers and preservatives should be explored and studied for use in the development of future formulations.

## Conclusion

Based on the literature data presented in this review, we suggest the following considerations in designing future hGH parenteral formulations with decreased ISP; First of all, The confounding effect of protein concentration and viscosity needs further investigation. Regarding the formulation’s tonicity, isotonic rhGH preparations with 300 mOsm/Kg are preferred (111). Formulations with higher osmolality (up to 600 mOsm/Kg) can be fairly tolerated. If the injection volume is less than 0.5 mL (114). Using Arginine-HCl is superior to NaCl for tonicity adjustment. If NaCl is necessary, it should be used in low concentrations (88). Sorbitol and mannitol can decrease ISP due to their local anesthetic effect (133–135).

Liquid preparations are preferred to lyophilized powder forms due to lower ISP and easier preparation steps before injection (54, 66, 77–81). Formulations which require less frequent injections are more desirable (64, 77, 100). For this purpose, the development of novel long-acting rhGH formulations is necessary (66–68). When frequent injections are necessary, injection volumes of less than 1 mL are preferred (89–92, 94, 95).

The product’s pH should be set close to 7.4 to decrease ISP (111, 119–121). Buffer-free formulations are superior to buffered rhGH products (88, 120). However, if buffers are necessary, low concentrations of low-strength buffers are preferred (88, 103, 120, 122–126). Avoiding the use of citrate in buffered formulations is also recommended (88, 116–118, 120, 124, 127). If citrate is necessary, incorporating Ca^2+^ ions or ASIC1 inhibitors in the formulation may negate the impact of citrate on ISP (120).

Regarding antimicrobial preservatives, avoiding the use of *m*-cresol in multi-dose preparations is advised (101, 102, 104–106). Instead, using benzyl alcohol is preferred due to its local anesthetic effect (107–110).

High-quality and highly pure surfactants can reduce injection pain (128). Using alternative surfactants such as alkylglucosides instead of PS20 and P188 is recommended (130, 131). Finally, letting the refrigerated rhGH formulations reach room temperature before administration is recommended (139, 143, 144).

## Author contributions

BT: conceptualization, writing-original draft, investigation. MJ: conceptualization, supervision. NZ: writing-review and editing, supervision. All authors contributed to the article and approved the submitted version.

## Conflict of interest

The authors declare that the research was conducted in the absence of any commercial or financial relationships that could be construed as a potential conflict of interest.

## Publisher’s note

All claims expressed in this article are solely those of the authors and do not necessarily represent those of their affiliated organizations, or those of the publisher, the editors and the reviewers. Any product that may be evaluated in this article, or claim that may be made by its manufacturer, is not guaranteed or endorsed by the publisher.
